# A case of virilzing adrenocortical carcinoma diagnosed by urine steroid profiling

**DOI:** 10.1186/1687-9856-2013-S1-P131

**Published:** 2013-10-03

**Authors:** Joanna Yuet-ling Tung, Angel OK Chan, Elim Man, Pik-to Cheung

**Affiliations:** 1 Department of Paediatrics and Adolescent Medicine, Queen Mary Hospital, The University of Hong Kong, Hong Kong; 2 Department of Pathology, Queen Elizabeth Hospital, Hong Kong

## Introduction

Childhood adrenocortical carcinoma is rare and has poor prognosis. Histopathological examination often helps in differentiating between benign versus malignant adrenal tumors. However, the diagnostic accuracy of adrenal biopsies varies and could be affected by the sampling and the quality of the specimen. Urine steroid profiling (USP) has been used to differentiate between malignant tumors from benign ones. We report a case of virilizing adrenocortical carcinoma in which the diagnosis was made with the help of the characteristic USP findings

## Case report

A 6-year old girl presented with left lower quadrant pain. Physical examination revealed a large abdominal mass of 9 x 9 cm in size. She has tall stature and deepened voice. There was no pubertal change except few pubic hairs and clitoromegaly. No gonads were palpable. There was no cushingoid features or hirsutism. Computed tomography showed a 12 cm well–defined heterogeneously-hypoenhancing mass at the upper pole of the left kidney. Ultrasound-guided biopsy of the tumor revealed necrotic tissue. Open biopsy showed fibroblastic tissue with no specific diagnosis. Luteinizing hormone, follicular stimulating hormone and testosterone were elevated for age at 1.7 IU/L, 6.1 IU/L and 1.8nmol/L respectively. Urine for free cortisol was normal. USP showed markedly increased metabolites of dehydroepiandrosterone. Tetrahydro-11-deoxycortisol and 3-alpha, 16-alpha, 20-alpha pregnenetriol, which are metabolites reported to be increased in malignant adrenocortical tumors, were also elevated. The findings were suggestive of a steroid-secreting malignant adrenocortical tumor, which predominantly synthesizes androgens. Laparotomy was done and an 11 x 10 cm well encapsulated tumor arising from the left adrenal gland was found. Complete removal of the tumor was performed. Histological exam showed an extensively necrotic adrenocortical carcinoma with no evidence of invasion into capsule, adjacent lymph nodes or nerves. USP was repeated 1 month post-operatively and showed no excessive excretion of androgen metabolites, which suggested complete tumor removal.

## Conclusion

Histopathological exam from tumor biopsy could be misleading in adrenocortical carcinoma due to sampling problem. On the other hand, hormonal overproduction and production of unusual metabolites could be revealed by USP and this helps in the diagnosis and follow-up of patients.

